# Protective Effects of *Lactobacillus plantarum* CCFM8246 against Copper Toxicity in Mice

**DOI:** 10.1371/journal.pone.0143318

**Published:** 2015-11-25

**Authors:** Fengwei Tian, Yue Xiao, Xiaoxiao Li, Qixiao Zhai, Gang Wang, Qiuxiang Zhang, Hao Zhang, Wei Chen

**Affiliations:** 1 State Key Laboratory of Food Science and Technology, School of Food Science and Technology, Jiangnan University, Wuxi, Jiangsu, People’s Republic of China; 2 Beijing Innovation Centre of Food Nutrition and Human Health, Beijing Technology & Business University, Beijing 100048, People’s Republic of China; University of British Columbia, CANADA

## Abstract

*Lactobacillus plantarum* CCFM8246, which has a relatively strong copper binding capacity and tolerance to copper ions, was obtained by screening from 16 lactic acid bacteria in vitro. The selected strain was then applied to a mouse model to evaluate its protective function against copper intoxication in vivo. The experimental mice were divided into an intervention group and a therapy group; mice in the intervention group received co-administration of CCFM8246 and a copper ion solution by gavage, while mice in the therapy group were treated with CCFM8246 after 4 weeks of copper exposure. In both two groups, mice treated with copper alone and that treated with neither CCFM8246 nor copper served as positive and negative controls, respectively. At the end of the experimental period, the copper content in feces and tissues, the activity of alanine aminotransferase (ALT) and aspartate aminotransferase (AST) in serum, and oxidation stress indices in liver and kidney tissue were determined. Learning and memory ability was evaluated by Morris water maze experiments. The results indicated that treatment with CCFM8246 significantly increased the copper content in feces to promote copper excretion, reduce the accumulation of copper in tissues, reverse oxidative stress induced by copper exposure, recover the ALT and AST in serum and improve the spatial memory of mice.

## Introduction

Copper pollution accidents have occurred frequently in recent years, which drew public concern on the problem of copper toxicity. Although copper is an essential trace element and its toxicity is far lower than that of other heavy metals, it can be toxic once the safe dose is exceeded. Ignorance about potential toxicity may increase the likelihood of damage due to copper contamination. Copper in water and soil can enter the food chain and may accumulate in humans and animals via ingestion of contaminated food and drinking water [1–3].Copper is mainly stored in the liver but can reach the brain, kidneys and other tissues and organs via the bloodstream[4]. As well as directly causing liver damage, excess copper in the blood can cause renal and brain damage and negatively influence learning and memory ability, as seen in Wilson’s disease[5]. The mechanisms of copper toxicity demonstrated in previous studies involve the inhibition of superoxide dismutase (SOD), glutathione peroxidase (GSH-PX) and other antioxidant enzymes and depletion of reduced glutathione (GSH), meaning that free radicals cannot be scavenged and lipid peroxidation and oxidative DNA damage is increased[6, 7].

The most direct way to alleviate copper intoxication is chelation therapy, which can promote copper excretion[8, 9]. However, chelators usually have some unavoidable side effects including renal toxicity and the loss of essential trace elements[10]. Previous reports have suggested that some lactobacilli can bind and remove heavy metals such as cadmium, lead, and copper in vitro [11–14]. Besides their copper binding capacity, the known antioxidative properties of lactobacilli may also be important [15, 16]. These two factors suggest that lactobacilli may have a potent protective action against copper toxicity. As far as we know, there are very few studies on the protective effects of lactobacilli against copper toxicity, and the corresponding protection mechanism has not been studied yet.

In this study, a lactobacilli with good copper binding and tolerance ability was selected and its protective effects against copper-induced damage in vivo were evaluated using mouse models.

## Materials and Methods

### Chemicals and reagents

Kits used to measure the levels of malondialdehyde (MDA) and the activities of superoxide dismutase (SOD), aspartate aminotransferase (AST), alanine aminotransferase (ALT) and glutathione peroxidase (GSH-PX) were purchased from the Jiancheng Bioengineering Institute (Nanjing, China). Kits used to measure the protein concentration were purchased from Thermo Scientific Pierce Company (America). Copper sulfate pentahydrate and other analytical laboratory chemicals and reagents were purchased from Sinopharm Chemical Reagent Company (Shanghai, China).

### Bacterial strains and culture

*Lactobacillus rhamnosus* GG and 15 other strains (*Lactobacillus plantarum* CCFM8246, CCFM39 and CCFM8610, *Lactobacillus casei* CCFM38, *Bifidobacterium* CCFM8614 and CCFM16, *Lactobacillus bulgaricus* CCFM24, *Lactobacillus acidophilus* CCFMLa, *Lactobacillus thermophilus* CCFM32, *Lactobacillus reuteri* CCFMLR and CCFM13, *Lactobacillus sake* CCFM14, *Lactobacillus helveticus* CCFML.hel, *Lactobacillus delbrueckii* CCFM2-3 and *Lactobacillus fermentum* CCFMZ7) were obtained from the Culture Collections of Food Microbiology, Jiangnan University (Wuxi, China). All strains were cultured in de Man, Rogosa and Sharpe (MRS) broth (Hopebio Company, Qingdao, China) at 37°C for 18 h.

### Copper binding experiments

After 18-hour culture, MRS broth containing each strain was centrifuged at 4000g, 4°C for 20 min and the supernatant was removed to collect the biomass. The collected pellet was washed three times with sterilized saline and then divided into two parts. One part was immediately used as living biomass. The other part was sterilized in a high pressure steam sterilizer at 121°C for 15 min serving as dead biomass. Before the binding experiment, the dry cell weight of both living and dead biomass was obtained via an oven According to the previous report[17, 18], a weighted amount of biomass (both living and dead cell pellets) was resuspended with ultrapure water containing 50 mg/liter copper ions to reach a final bacterial concentration of 1 g dry biomass/liter and the pH of the suspension was immediately adjusted to 6.0. The samples were incubated at 28°C, 150 r/min for 12 h in a thermostatic incubator shaker and then centrifuged at 4000g for 20 min. Residual copper in the supernatant was analyzed by flame atomic absorption spectrophotometry (Spectra AA 220; Varian). The original solution containing 50 mg/liter copper ions served as the blank control. The amount of copper uptake *q* (mg/g) was calculated using the following mass balance equation:

$$q=\left(C_{0}-C_{l}\right)V/m$$

where C_0_ (mg/L) is the initial copper ion concentration, C_l_ (mg/L) is the copper ion concentration after adsorption, V (L) is the volume of the solution and m(g) is the amount of biomass.

### Copper tolerance experiments

The strains were inoculated into a series of MRS broths with different concentrations of copper ions (0 mg/L, 50 mg/L, 150 mg/L, 500 mg/L) at an inoculum level of 2% (v/v). During the 25-hour cultivation, a group of samples was taken every 2 hours and absorbance at 600 nm measured to detect bacterial growth.

### Assays for tolerance of artificial gastric juice and bile salts

The collected biomass was washed twice with sterilized saline and then resuspended. The resuspended liquid was mixed with artificial gastric juice (0.3%pepsin and 0.2% sodium chloride adjusted to pH 2.5 with HCl and then sterilized by filtration) at a volume ratio of 1:9 (1 mL: 9 mL) in a test tube, with an equal volume of saline serving as the control. The test tube was incubated at 37°C for 3 h. During this period, the tube was turned up and down every 30 min to keep the phase homogeneous. Bile salts were added to the MRS broth to reach a series of final concentrations from 0 to 0.4%. Inoculum was added at 2% (v/v) and the cultures were incubated at 37°C for 20 h. After incubation, the optical density of the culture liquid was measured at 600 nm to detect growth of lactobacilli.

### Animals and experimental design

Adult male C57black/6 mice used in all of the experiments were obtained from the Shanghai Laboratory Animal Center (Shanghai, China). Mice were selected strictly by weight (between 28 and 30 g) and age (around 8 weeks old).Mice were kept in stainless steel cages in a temperature- and humidity-controlled room that was able to maintain a 12 h light/dark cycle. Mice were fed with standard commercial mouse food and water was given ad libitum. All of the protocols for this study were approved by the Ethics Committee of Jiangnan University, China (JN no. 20140509-0627(16)). All procedures were carried out in accordance with the European Community guidelines (Directive 2010/63/EU) for the care and use of experimental animals.

Skimmed milk (12% w/w) was added to the collected biomass serving as protectant. The cell pellet was freeze-dried to obtain the strain powder. The concentration of lactobacilli was finally adjusted to 2×10^10^CFU/mL.

#### Protective effects of the selected strain against copper exposure

As is shown in Table 1, Mice were randomly divided into two major groups. Intervention groups were used to investigate the effect of co-administration of lactobacilli and copper, while therapy groups were used to study the therapeutic function of lactobacilli after copper toxicity had been established. Each of these two major groups was divided into four subgroups. For intervention groups, the experimental period was 4 weeks. Mice received lactobacilli after they had been exposed to copper for 1 h. For the therapy groups, the experimental period was 8 weeks.

**Table 1:** Animal experimental protocol.

Treatment on the indicated weeks for the following:		
	1–4	5–8
**1**	SM (CK)	SM
**2**	Cu(100)+SM (I)	—
**3**	Cu(150)+SM (I)	—
**4**	Cu(100)+8246+SM (I)	—
**5**	Cu(150)+8246+SM (I)	—
**6**	Cu(100)+SM (T)	SM
**7**	Cu(150)+SM (T)	SM
**8**	Cu(100)+SM (T)	8246+SM
**9**	Cu(150) +SM (T)	8246+SM

SM, 0.2 mL skimmed milk by gavage ODAT; Cu (100), the concentration of CuSO_4_ given once daily by gavage was 100 mg/kg mouse weight; Cu (150), the concentration of CuSO_4_ given once daily by gavage was 150 mg/kg mouse weight; 8246+SM, 0.2 mL skimmed milk by gavage ODAT which contains a concentration of 2×10^10^CFU/mL lactobacilli CCFM8246.There are 10 mice in each of 9 groups; CK, the control group; I, the intervention group; T, the therapy group.

Mice in both the intervention and therapy groups were given 0.2 mL skimmed milk containing 2×10^10^CFU/mL lactobacilli by gavage once daily. The doses and periods of administration of lactobacilli were based on the previous study[19,20].As previously reported[21], the toxic dose of copper for mice was designated as 100 mg/kg mouse weight and 150 mg/kg mouse weight once daily.

During the experimental period, each mouse was moved into a clean, empty cage every week for 1 h and fecal samples were collected. There was no death of mice during the whole experiments. At the end of experiment mice were sacrificed under light ether anesthesia and blood was collected in heparinized tubes to obtain plasma. The liver, kidneys and brains were excised and washed with saline solution. The remaining liver, kidney and brain tissues were collected in metal-free Eppendorf tubes and stored at -80°C for biochemical assays and estimation of copper concentration. Levels of copper were analyzed by flame atomic absorption spectrophotometry (Spectra AA 220; Varian). In order to avoid the contamination of any other metal, feces and tissues were collected in clean Eppendorf tubes, which are made of polypropylene. Before the measurement of metals, tissues and feces were transferred to digestion vessels and digested in concentrated HNO_3_ using the Microwave Digestion System. The digestion vessels have TFM-wetted surfaces and were washed with ultrapure water thoroughly before use. The levels of MDA and the activities of SOD, GSH-PX, ALT and AST were measured by using assay kits purchased from the Jiancheng Bioengineering Institute (Nanjing, China), the concrete protocols were according to the recommendations of the manufacturer.

#### Morris water maze experiments

A modified version of Liu Li’s experimental method was used[22]. The Morris water maze apparatus was divided into four quadrants and a hidden platform was placed in the target quadrant.

The training and test was conducted a week before the end of the experiment. During the first 4 days, mice were trained with a hidden platform. The time that each mouse spent seeking the submerged platform was controlled by the observer. Four trials were conducted each day, for 4 consecutive days.

On the day 5, the platform was removed and the probe test was conducted. The spatial memory was evaluated according to the time the mouse swam in the target quadrant.

### Data processing and statistical analysis

Differences between groups were analyzed using one-way analysis of variance in SPSS 20.0, followed by Tukey’s *post hoc* test. Data were expressed as the mean±the standard error of the mean (SEM) for each group. A *p* value < 0.05 was considered to be statistically significant.

## Results and Discussion

### Screening of Lactobacilli with copper binding capacity and tolerance

#### Copper binding experiments

**Fig 1:**
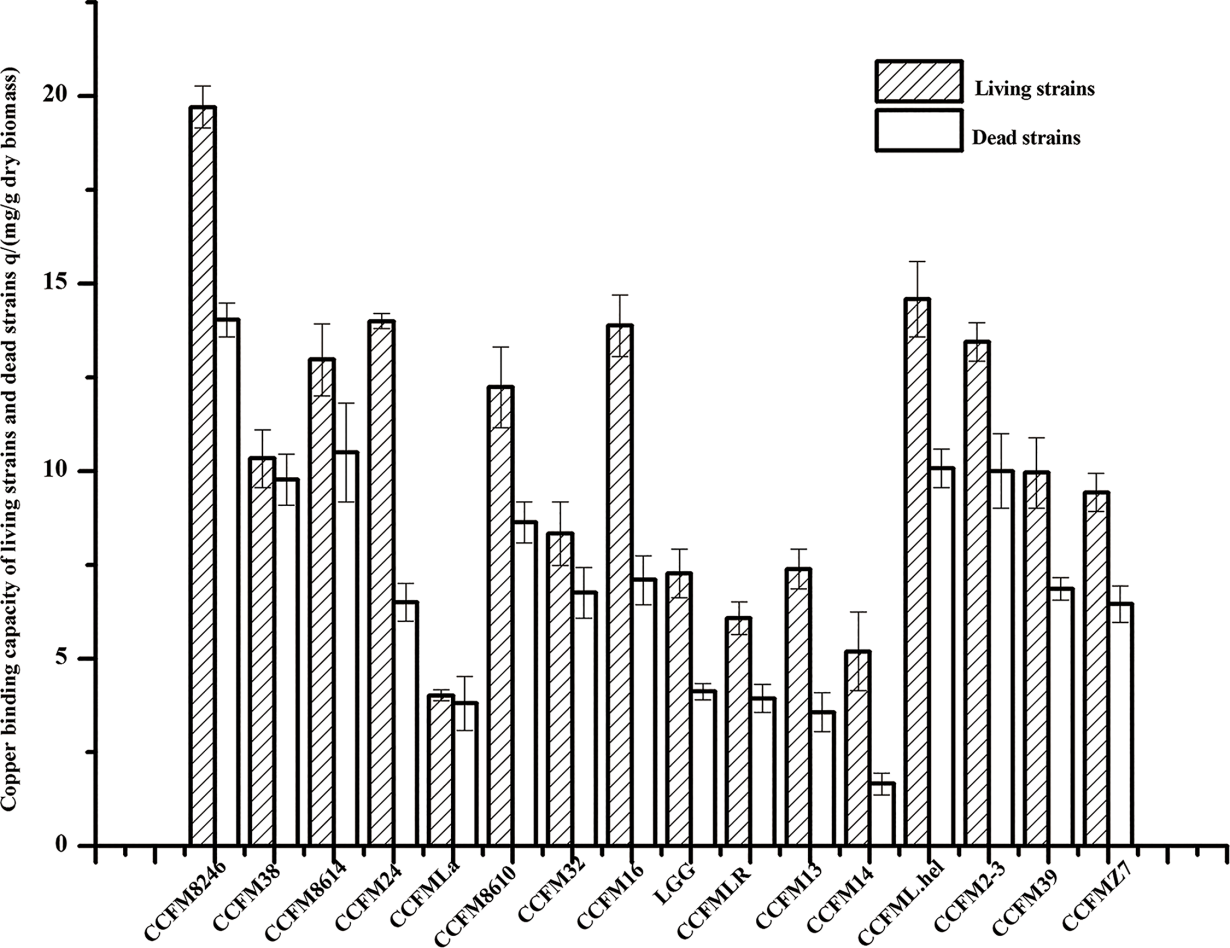
Copper binding capacity of bacterial strains. The average of three replicates and standard deviation are presented. CCFM8246, CCFM8614 and CCFM8610 are *Lactobacillus plantarum*. CCFM38 is *Lactobacillus casei*. CCFM24 is *Lactobacillus bulgaricus*. CCFMLa is *Lactobacillus acidophilus*. CCFM32 is *Lactobacillus thermophilus*. CCFM39 and CCFM16 are *Bifidobacterium*. LGG is *L*. *rhamnosus* GG. CCFMLR and CCFM13 are *Lactobacillus reuteri*. CCFM14 is *Lactobacillus sake*. CCFML.hel is *Lactobacillus helveticus*. CCFM2-3 is *Lactobacillus delbrueckii*. CCFMZ7 is *Lactobacillus fermentum*.

The copper binding capacities of 16 strains for both living and dead samples are shown in Fig 1. The binding capacities of the 16 strains fluctuated widely and were significantly influenced by the treatment of boiling. The biosorption levels of living strains were all higher than those of the dead strains, which corresponds to the findings of previous studies [19,20,23]. Among all the tested strains, *L*. *plantarum* CCFM8264 had the highest binding capacity of both living and dead groups, with adsorption values of 19 and 14 mg/g dry biomass, respectively.

The copper biosorption of lactobacilli might relate to physical adsorption and chemical adsorption, and ion exchange on the surface the bacteria is also likely to be a factor[13, 24, 25]. Judging from the experiments *in vitro*, there was a significant difference in copper binding capacity among different strains, which might be attributable to differences in structure, functional groups and species. The living bacteria had higher copper binding capacities than the dead ones, which might be due to that boiling treatment damaged cell structure irreversibly and reduced metal ion binding sites.

#### Copper tolerance experiments

**Fig 2:**
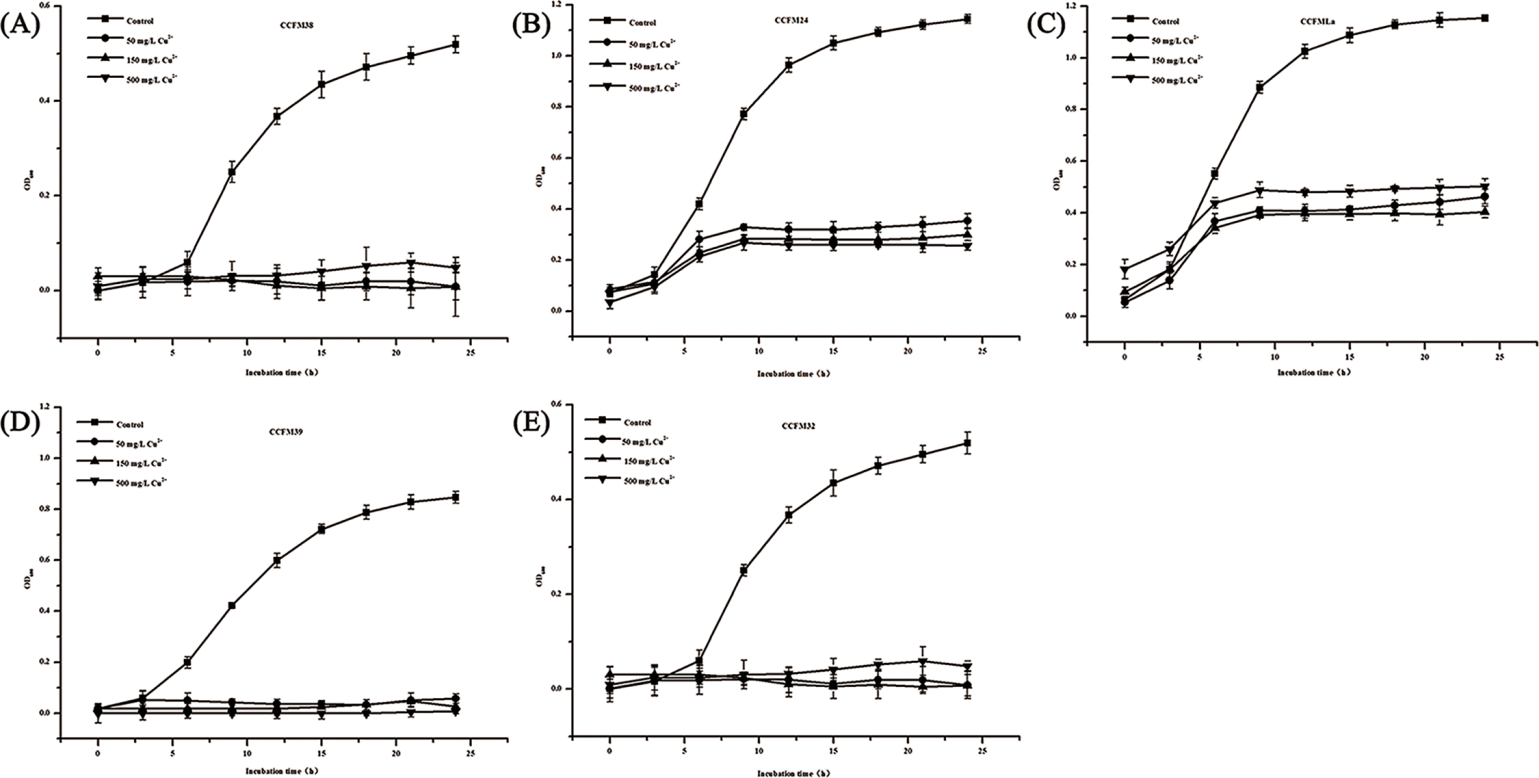
The first kind of growth of strains in different copper ion MRS broths. The subgraphs (A) to (E) show the tolerance ability of CCFM38, CCFM24, CCFMLa, CCFM39 and CCFM32, respectively.

**Fig 3:**
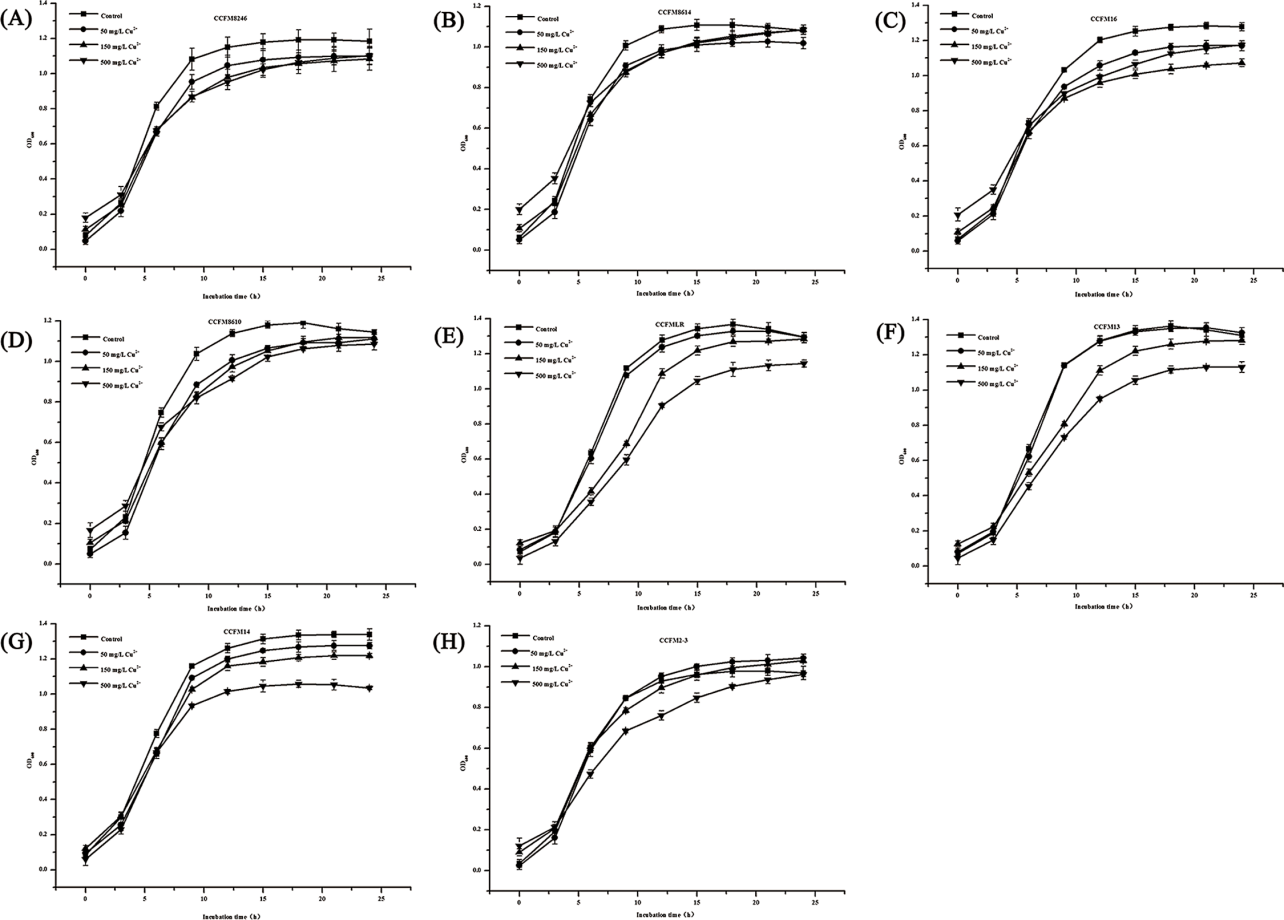
The second kind of growth of strains in different copper ion MRS broths. The subgraphs (A) to (H) show the tolerance ability of CCFM8246, CCFM8614, *Bifidobacterium*, CCFM8610, CCFMLR, CCFM13, CCFM14 and CCFM2-3, respectively.

**Fig 4:**
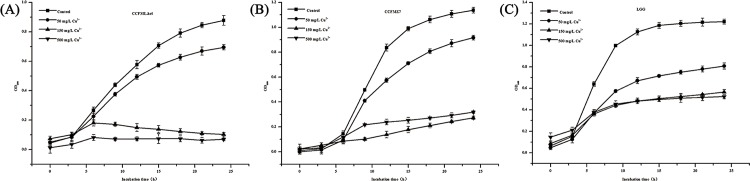
The third kind of growth of strains in different copper ion MRS broths. The subgraphs (A) to (C) show the tolerance ability of CCFML.hel, CCFMZ7 and LGG, respectively.

The growth curves of the 16 strains in MRS broths containing different concentrations of copper ions (0, 50, 150 and 500 mg/L) are shown in Figs 2–4. The growth patterns can be roughly divided into three types. The first type of growth curves are shown in Fig 2. These 5 strains were very sensitive to copper and generally could not grow in MRS broth containing copper ion concentrations of 50, 150 or 500 mg/L. The second type of growth curves are shown in Fig 3. No obvious inhibition of growth was observed in these strains. In addition, the growth tendencies of these strains were similar in broth containing different concentrations of copper ions. The third type of growth curves are shown in Fig 4. The growth of these strains was affected by different concentrations of copper ion to different degrees.

The results of the copper binding experiments and tolerance experiments were combined and the most promising strain, CCFM8246, which had the highest copper binding capacity and could also effectively tolerate copper inhibition, was selected for use in the subsequent experiments.

### Tolerance of *L*. *plantarum* CCFM8246 to artificial gastric juice and bile salts

The beneficial effects of lactic acid bacteria can be realized if the bacteria are able to survive in the gastrointestinal tract[20, 26] Therefore, for the development and production of useful products, the ideal lactic acid bacteria should be tolerant of human gastric juice and bile salts. The changes of viable cell counts after the treatment with artificial gastric juice at pH 2.5 for 3 h are shown in Table 2. Viable cell counts of *L*. *plantarum* CCFM8246 after 3 h in cultivation decreased by less than one order of magnitude and the strain could maintain a relatively high survival rate.

**Table 2:** Effects of artificial gastric juices on the viability of *L*. *plantarum* CCFM8246.

Viable cell counts (log CFU/mL)	
Control group (0 h)	Experimental group (3 h)
9.3±0.04	8.7±0.03^a^

^a^The letter a means that two groups differ significantly (P<0.05).

The content of bile salts in the human intestine is 0.03–0.30%. Only if lactic acid bacteria can grow and metabolize in physiologic concentrations of bile salts will they be able to survive in the intestine. As shown in Table 3, the highest concentration of bile salts that *L*. *plantarum* CCFM8246 could tolerate and grow in was 0.2%, which indicates that the strain would be well able to tolerate bile salts in the human intestine.

**Table 3:** The growth of *L*. *plantarum* CCFM8246 in bile salts of different concentration.

	Bile salts concentration				
	0.0%	0.1%	0.2%	0.3%	0.4%
**Growth condition**	++	++	+	−	−

++, CCFM8246has grown well in given bile salts concentration, the MRS liquid culture medium is turbid and megascopic cell precipitation can be clearly observed; +, CCFM8246 has grown slightly, the MRS liquid culture medium is turbid to a small extent and a little megascopic cell precipitation could be observed; −, CCFM8246 cannot grow, the culture medium is clear and transparent and no cell precipitation could be observed.

### Protective effects of CCFM8246 against copper exposure in mice

#### Effects of CCFM8246 on the levels of copper in mice feces

**Fig 5:**
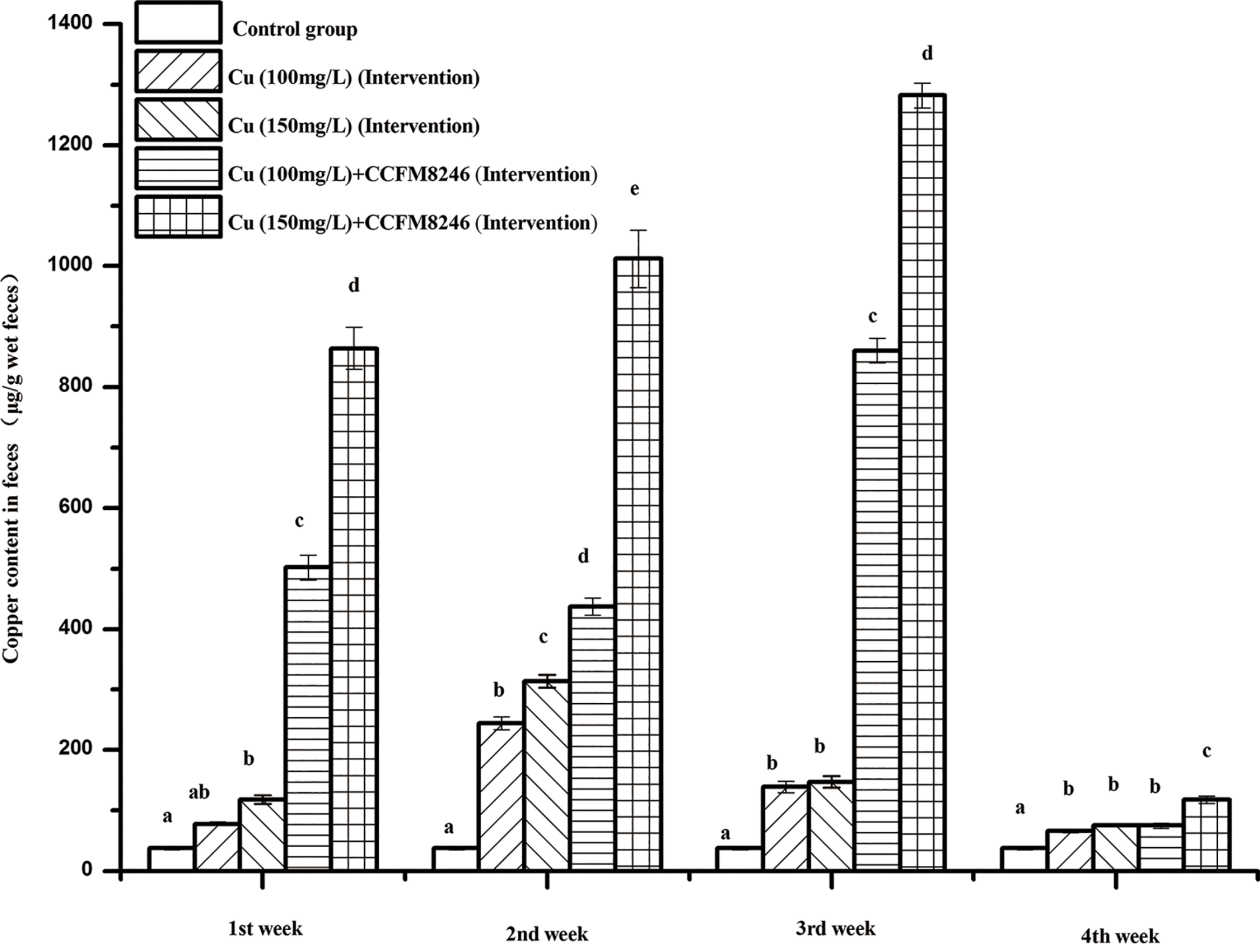
Copper content in wet feces of mice in intervention groups. Values are mean ± SEM with 10 mice in each group. The letters a, b, c, d and e mean that in each period, groups with different letters differ significantly (*p* <0.05).

**Fig 6:**
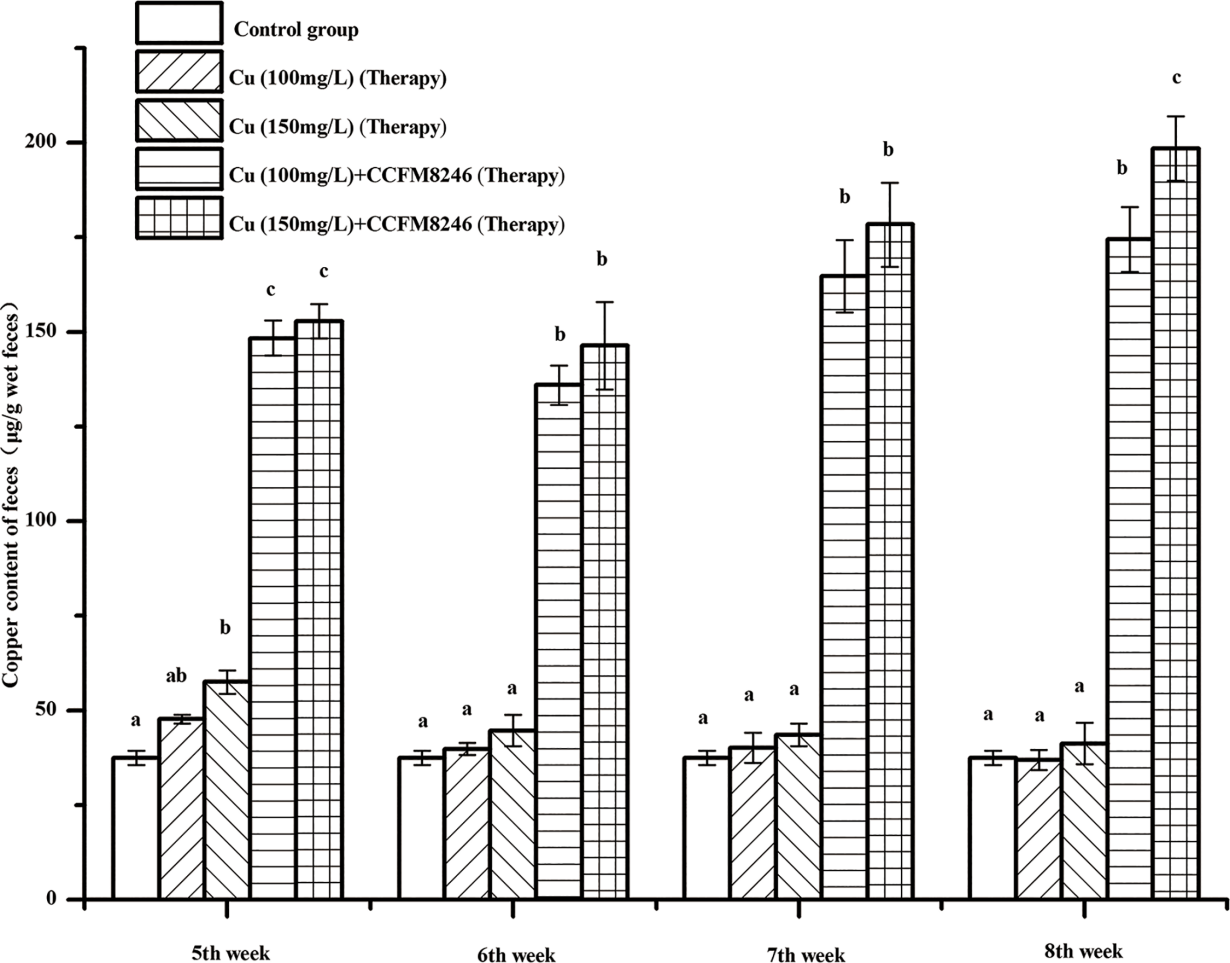
Copper content in wet feces of mice in therapy groups. Values are mean ± SEM with 10 mice in each group. The letters a, b and c mean that in each period, groups with different letters differ significantly (*p* <0.05).

The effects of CCFM8246 on levels of copper in mice feces are shown in Fig 5 and Fig 6 for intervention groups and therapy groups, respectively. In the intervention groups, it was observed that in the 4-week experimental period, the copper levels of feces in copper-only groups (the copper toxicity modeling groups) were both significantly lower than the copper levels in copper-plus-CCFM8246 groups (*p* <0.05), except for the results of the 100 mg/L copper group in the week 4. In the therapy groups, it was observed that during weeks 5 to 8, after stopping copper gavage, the copper levels in the feces of mice given CCFM8246 were significantly higher than those in the copper-only mice’s for the two copper ion concentrations (*p* <0.05). In addition, from week 6 to week 8 there was no significant change in the copper levels in the feces of the copper-only mice, which indicated that although copper exposure was ceased, mice still could not excrete copper spontaneously and needed the external auxiliary treatments to alleviate the damage from copper exposure.

According to previous reports, *Lactobacillus rhamnosus* ATCC 53013 can reduce the intestinal absorption of aflatoxin in rats and simultaneously promote its excretion via feces[27]. Judging from the results of the two groups, the CCFM8246 has a similar function and could quickly and effectively combine with a large number of copper ions in the body, reduce the intestinal absorption of copper ions and prompt excretion of copper ions via the feces.

#### Effects of CCFM8246 on the levels of copper in livers, kidneys and brains of copper-exposed mice

**Fig 7:**
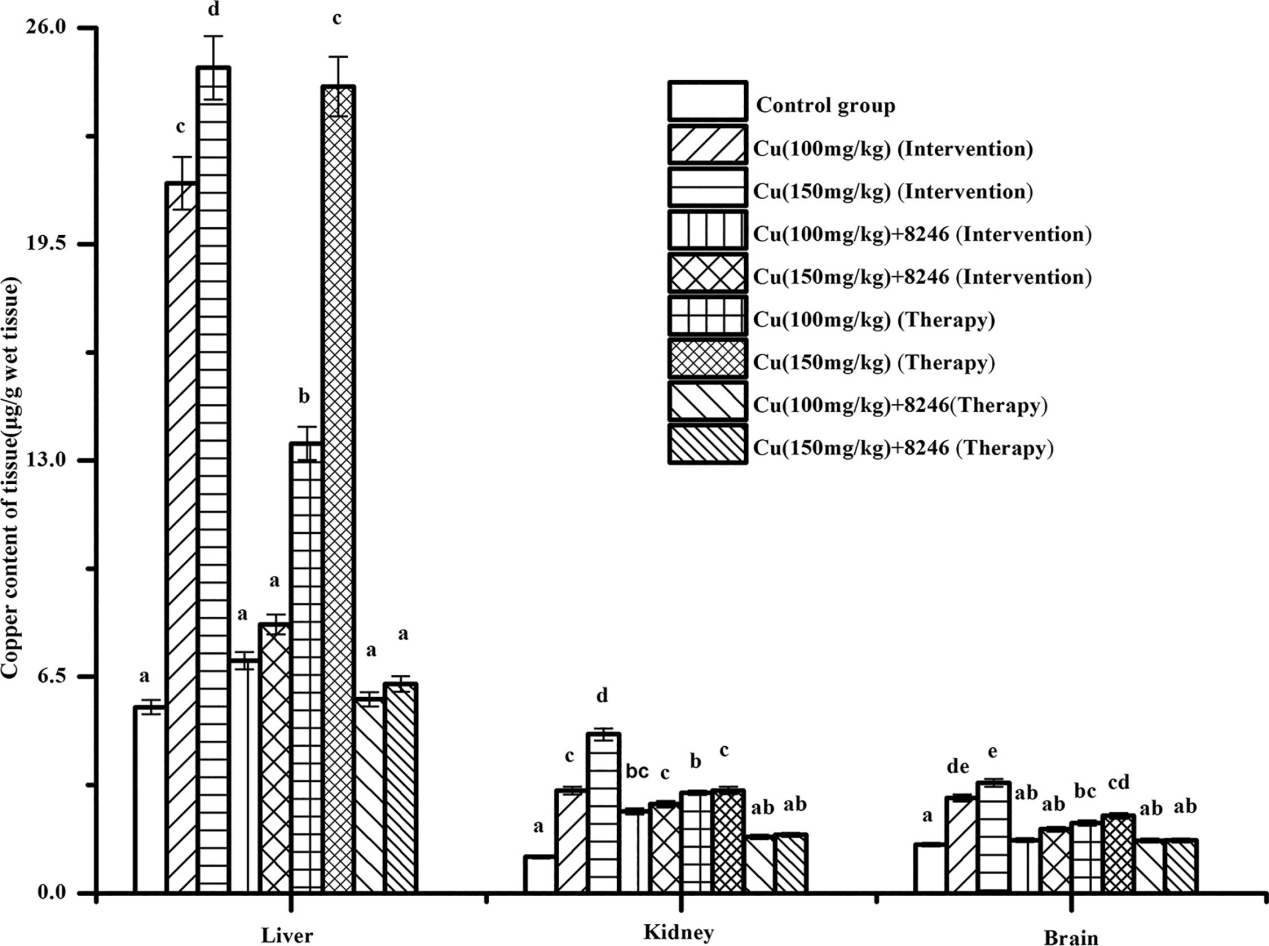
Copper content in livers, kidneys and brains of mice. Values are mean ± SEM with 10 mice in each group. The letters a, b, c, d and e mean that in each tissue, groups with different letters differ significantly (*p* <0.05).

The effects of CCFM8246 on levels of copper in the livers, kidneys and brains of copper-exposed mice in both intervention groups and therapy groups are shown in Fig 7. The levels of copper in these three organs of the copper-only mice were significantly higher than those in the control group (*p* <0.05). The copper content in these three organs of the copper toxicity modeling mice given CCFM8246 for intervention or therapy was lower than in the corresponding-dose copper toxicity modeling groups (*p* <0.05). These results indicate that the strain CCFM8246 could indeed reduce the copper content in copper-exposed mice, which further verified its function of promoting copper excretion by feces.

#### Effects of CCFM8246 on the intelligence of copper-exposed mice

As shown in Table 4, through training, mice learned the spatial orientation and memory of the location of the submerged platform. The average escape latency of the copper-only mice was prolonged significantly compared with the control group (*p* <0.05), which suggested that they suffered intelligence damage and copper exposure impaired learning and memory. According to previous reports, copper exposure causes a decrease in the spatial learning and memory ability of mice; the mechanism might be related to oxidative stress in the brain caused by excessive copper ions, causing damage in the cerebral cortex and nervous system[28]. The escape latencies of the CCFM8246 treated mice in both the intervention and therapy groups were significantly shortened after the second day compared with mice exposed to copper with no intervention or therapy (*p* <0.05).

**Table 4:** The results of Morris water maze experiment (navigation training for spatial orientation).

Treatment	Escape latency(s)			
	Day 1	Day 2	Day 3	Day 4
**——-(CK)**	51.99±1.00^a^	40.63±0.57^a^	27.71±0.26^a^	20.38±1.47^a^
**Cu(100)(I)**	54.32±1.06^c^	51.27±0.64^c^ ^d^	46.23±1.07^d^	40.21±2.03^e^
**Cu(100)+8246(I)**	54.58±0.52^c^	50.77±1.57^b^ ^c^ ^d^	40.60±0.53^b^	33.27±0.74^d^
**Cu(150)(I)**	54.83±1.26^c^	52.17±2.02^c^ ^d^	48.20±0.72^d^	42.67±2.08^e^
**Cu(150)+8246(I)**	53.67±1.53^a^ ^b^ ^c^	50.47±1.29^d^	41.10±0.36^b^	36.83±3.01^d^
**Cu(100)(T)**	53.53±0.50^a^ ^b^ ^c^	50.67±2.08^b^ ^c^ ^d^	44.90±4.26^c^ ^d^	33.98±1.00^c^ ^d^
**Cu(100)+8246(T)**	52.70±0.27^a^ ^b^	48.63±0.65^b^	39.20±2.88^b^	27.12±2.51^b^
**Cu(150)(T)**	53.77±0.68^b^ ^c^	51.10±1.15^c^ ^d^	47.17±1.62^d^	34.73±0.93^c^ ^d^
**Cu(150)+8246 (T)**	53.93±0.74^b^ ^c^	49.60±0.56^b^ ^c^	41.90±0.17^b^ ^c^	28.10±1.01^b^

Values are mean ± SEM with 10 mice in each group; CK, the control group; I, the intervention group; T, the therapy group.

^a,b,c,d,e^The letters a, b, c, d and e mean that on each day, groups with different letters differ significantly (P<0.05).

As shown in Table 5, compared with the control groups, the times of navigating across the original location of the platform, the ratio of navigation time in the targeted quadrant and the ratio of navigation distance in the targeted quadrant of only-copper-exposed mice were all significantly decreased (*p* <0.05), indicating that mice’s memory of the original location of the platform was still not impressed after the four-day training. However, compared with only-copper-exposed mice, each index of CCFM8246 treated mice was increased (*p* <0.05). These results indicate that although copper exposure caused the memory of mice to decline, this symptom could be effectively alleviated with CCFM8246 treatment.

**Table 5:** The results of Morris water maze experiment (spatial exploration experiments).

Treatment	Indices of spatial exploration		
	Times of crossing	Time ratio	Distance ratio
**——-(CK)**	3.30±0.82^c^	38.20±2.03^e^	34.44±0.98^d^
**Cu(100)(I)**	1.10±0.58^a^	16.10±0.85^c^	15.21±1.54^a^
**Cu(100)+8246(I)**	2.50±0.71^b^	25.69±3.05^d^	29.77±1.36^c^ ^d^
**Cu(150)(I)**	0.50±0.53^a^	13.33±1.43^b^ ^c^	15.67±0.58^a^
**Cu(150)+8246(I)**	2.40±0.20^b^	24.33±3.06^d^	27.33±1.36^b^ ^c^
**Cu(100)(T)**	0.90±0.74^a^	9.60±0.53^a^	18.30±1.47^a^
**Cu(100)+8246(T)**	2.40±0.84^b^	15.44±1.50^c^	27.33±1.15^b^ ^c^
**Cu(150)(T)**	0.80±0.79^a^	11.17±1.26^a^ ^b^	15.67±2.08^a^
**Cu(150)+8246(T)**	2.00±0.67^b^	14.67±2.08^b^ ^c^	24.33±1.53^b^

Times of crossing, the times of mice navigating across the original location of the platform within 1 min; Time ratio, the ratio of navigation time in the quadrant where the platform was located (the swimming time in original-platform-located quadrant/total swimming time); Distance ratio, the ratio of navigation distance in the quadrant where the platform was located (the swimming distance in original-platform-located quadrant/total swimming distance).Values are mean ± SEM values with 10 mice in each group; CK, the control group; I, the intervention group; T, the therapy group.

^a,b,c,d,e^The letters a, b, c, d and e mean that in each index, groups with different letters differ significantly (*p* <0.05).

#### Effects of CCFM8246 on oxidative stress indices in the livers and kidneys of copper-exposed mice

The activity of SOD and GSH-PX and levels of MDA in mice livers and kidneys are shown in Table 6 and Table 7, respectively. Copper exposure led to a marked decline of the activity of SOD and simultaneously increased the levels of MDA. Compared with the copper-only groups, the activity of SOD and GSH-PX in mice livers, which are considered the crucial organs of copper storage, increased significantly after CCFM8246 administration with a simultaneous decrease in MDA levels (*p* <0.05). Meanwhile, for the kidneys containing relatively lower amounts of copper ions, most indicators were also significantly different after CCFM8246 intervention and therapy. These results show that copper exposure could induce oxidative damage and CCFM8246 had protective effects against such damage to some extent.

**Table 6:** Effects of CCFM8246 treatment on the activity of SOD, GSH-PX and level of MDA in the livers of mice.

Treatment	Oxidation stress indices		
	SOD	GSH-PX	MDA
**——-(CK)**	110.59±10.50^d^	202.07±16.95^e^	9.74±2.14^a^
**Cu(100)(I)**	59.18±5.01^b^	164.92±8.53^a^ ^b^ ^c^	20.02±3.55^d^ ^e^
**Cu(150)(I)**	41.15±4.01^a^	149.71±10.53^a^	23.08±1.90^e^
**Cu(100)+8246(I)**	94.53±7.41^c^	189.17±6.05^d^ ^e^	14.86±1.88^b^
**Cu(150)+8246(I)**	88.30±7.75^c^	173.55±7.30^b^ ^c^ ^d^	16.09±2.01^b^ ^c^ ^d^
**Cu(100)(T)**	66.19±5.01^b^	161.31±7.65^a^ ^b^	19.53±2.45^c^ ^d^ ^e^
**Cu(150)(T)**	45.03±3.31^a^	147.43±6.36^a^	22.80±2.03^e^
**Cu(100)+8246(T)**	91.03±3.35^c^	185.16±15.54^c^ ^d^ ^e^	14.55±1.65^b^
**Cu(150)+8246(T)**	86.91±6.31^c^	175.22±14.95^b^ ^c^ ^d^	15.63±2.15^b^ ^c^

Values are mean ± SEM values with 10 mice in each group; CK, the control group; I, the intervention group; T, the therapy group. The units for columns of SOD, GSH-PX and MDA are U/mg protein, U/mg protein and nmol/mg protein.

^a,b,c,d,e^The letters a, b, c, d and e mean that in each index, groups with different letters differ significantly (*p* <0.05).

**Table 7:** Effects of CCFM8246 treatment on the activity of SOD, GSH-PX and level of MDA in the kidneys of mice.

Treatment	Oxidation stress indices		
	SOD	GSH-PX	MDA
**——-(CK)**	22.72±3.04^d^	190.37±19.65^d^	13.42±1.51^a^
**Cu(100)(I)**	15.64±1.66^a^ ^b^	153.59±17.44^a^ ^b^	20.42±2.42^b^
**Cu(150)(I)**	12.10±0.85^a^	147.12±8.95^a^	29.92±3.04^d^
**Cu(100)+8246(I)**	19.67±2.30^c^ ^d^	172.41±10.13^b^ ^c^	15.12±1.02^a^
**Cu(150)+8246(I)**	17.63±1.96^b^ ^c^	166.24±8.11^b^ ^c^	16.59±1.41^a^ ^b^
**Cu(100)(T)**	15.37±3.81^a^ ^b^	151.39±14.42^a^ ^b^	19.67±3.09^b^
**Cu(150)(T)**	14.93±1.25^a^ ^b^	148.80±12.41^a^ ^b^	24.47±2.46^c^
**Cu(100)+8246(T)**	20.07±1.15^c^ ^d^	179.85±6.92^d^	15.41±1.63^a^
**Cu(150)+8246(T)**	18.85±1.36^b^ ^c^	169.67±11.50^b^ ^c^	17.38±2.48^a^ ^b^

Values are mean ± SEM values with 10 mice in each group; CK, the control group; I, the intervention group; T, the therapy group;The units for columns of SOD, GSH-PX and MDA are U/mg protein, U/mg protein and nmol/mg protein.

^a,b,c,d,e^The letters a, b, c, d and e mean that in each index, groups with different letters differ significantly (*p* <0.05).

#### Effects of CCFM8246 on activity of ALT and AST in serum of copper-exposed mice

**Fig 8:**
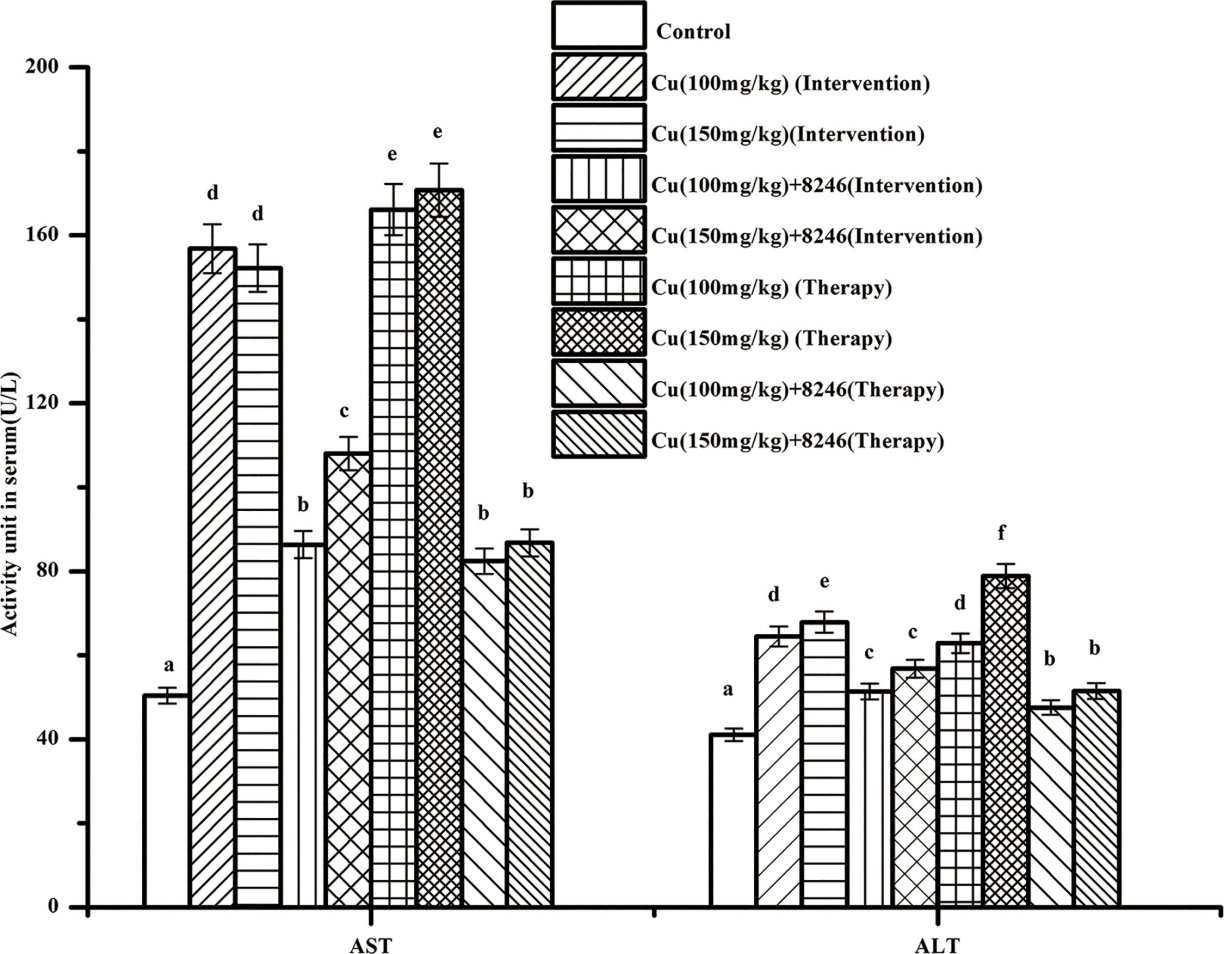
The activity of ALT and AST in serum of mice. The letters a, b, c, d, e and f mean that for each index, groups with different letters differ significantly (*p* <0.05).

AST and ALT are specific markers of hepatic damage [29, 30]. As shown in Fig 8, compared with the control groups, the activity of ALT and AST in the serum of copper-only mice was significantly elevated (*p* <0.05), indicating that copper toxicity did cause liver damage. However, compared with copper-only mice, the activity of ALT and AST in the serum of mice treated with CCFM8246 was significantly decreased (*p* <0.05), indicating that the intervention and therapy with CCFM8246 did alleviate the liver damage caused by copper exposure.

In conclusion, this study showed that *L*. *plantarum* CCFM8246 has protective function against acute copper toxicity in mice. It is the first time that the effects of lactobacilli on reducing intestinal copper absorption, decreasing tissue copper accumulation, ameliorating renal and hepatic oxidative stress, and alleviating hepatic damage are revealed. These interesting results demonstrated that *L*. *plantarum* CCFM8246 might have the potential to be a supplementary element to provide a novel dietary therapeutic strategy against acute copper toxicity.
